# Inter-floor noise classification using convolutional neural network

**DOI:** 10.1371/journal.pone.0243758

**Published:** 2020-12-22

**Authors:** Hye-kyung Shin, Sang Hee Park, Kyoung-woo Kim

**Affiliations:** Department of Living and Built Environment Research, Korea Institute of Civil Engineering and Building Technology, Goyang-Si, Kyeonggi-Do, Korea; Torrens University Australia, AUSTRALIA

## Abstract

In apartment houses, noise between floors can disturb pleasant living environments and cause disputes between neighbors. As a means of resolving disputes caused by inter-floor noise, noises are recorded for 24 hours in a household to verify whether the inter-floor noise exceeded the legal standards. If the noise exceeds the legal standards, the recorded sound is listened to, and it is checked whether the noise comes from neighboring households. When done manually, this process requires time and is costly, and there is a problem of whether the listener’s judgments of the sound source are consistent. This study aims to classify inter-floor noise according to noise sources by using a convolutional neural network model. A total of 1,515 sound sources of data recorded for 24 h from three households were annotated, and 40 4s audio clips of six noise sources, including “Footsteps,” “Dragging furniture,” “Hammering,” “Instant impact (dropping a heavy item),” “Vacuum cleaner,” and “Public announcement system” were identified. Moreover, datasets of 16 classes using ESC50’s urban sound category audio were used to distinguish the inter-floor noise heard indoors from the external noise. Although DenseNet, ResNet, Inception, and EfficientNet are models that use images as their domains, they showed an accuracy of 91.43–95.27% when classifying the inter-floor noise dataset. Among the reviewed models, ResNet showed an accuracy of 95.27±2.30% as well as a highest performance level in the F1 score, precision, and recall metrics. Additionally, ResNet showed the shortest inference time. This paper concludes by suggesting that the present findings can be extended in future research for monitoring acoustic elements of indoor soundscape.

## Introduction

The noise transmitted owing to the behavior of a neighbor in an apartment building is a factor that impedes a pleasant living environment [1–3]. Many parts of the world, including the EU, suggest sound insulation performance as a building requirement [4–6], and efforts are being made to resolve conflicts caused by noise between neighbors [7–9]. Noise from neighboring apartments not only causes residents’ stress but has also been found to lead to more extreme outbursts, such as murder, arson, and other forms of physical violence, owing to exaggerated conflicts between neighbors [10]. In the case of South Korea, the main noise source of neighbor noise is the impact sound generated by running or walking, accounting for 70.6% of all neighbor noise complaints, followed by hammering with 4.1%, dragging furniture with 3.4%, and household appliances (TV, vacuum cleaner, and washing machine) with 3.4% [11].

As a means to mediate disputes caused by inter-floor noise, consultation visits and noise measurement services can be used [11]. A noise measurement service records the noise heard in the lower unit for one day (24 h) and then determines whether the noise exceeding the legal standard is generated by the neighboring household. The noise measurement results are used by the dispute mediation committee as the basis for determining whether it has exceeded the inter-floor noise standard and to calculate the compensation amount [9]. This method requires time and cost to determine the noise source, after listening to all recorded data manually. There is also the question of whether data measured for one day is sufficient to be representative of the inter-floor noise of the corresponding household. Further, there is a possibility of privacy infringement. There is also a need to study whether the judgment about the noise source is objective and reproducible. For these reasons, less than 4.0% of people who file complaints use this service [11].

Studies on subjective responses because of inter-floor noise have been conducted mainly under laboratory conditions. Jeon et al. analyzed the annoyance caused by a heavy impact noise generated by a rubber ball and organized heavy impact noise levels into five classes according to annoyance [12]. Lee et al. analyzed the relationship between the subjective response to the heavy impact sound generated by the rubber ball, and evaluated the floor impact sound level (L_Amax_) as a practical descriptor that corresponds well to subjective response [13]. Yeon et al. studied the correlation between the noise level of various real impact sources and the single number quantities of standard heavy impact noise [14]. However, subjective response to noise is affected not only by physical factors of noise but also by non-acoustic factors and personal characteristics and is dependent on the context [15, 16]. Park and Lee examined non-acoustic factors affecting the reaction to impact noise [17]. And noise sensitivity and house ownership were found to have an impact on subjective responses. Yu and Kang considered physical, behavioral, social, demographical, psychological, and acoustic aspects of the soundscape response in urban space [18]. A study on a model that predicts subjective response by comprehensively considering both acoustic and non-acoustic factors is needed in the field of inter-floor noise. For such model study, acoustic factors, such as noise sources, levels, and occurrence times, need to be quantified by monitoring the inter-floor noise in real living environments for a long period.

To monitor the noise over a long period of time, a system that automatically classifies the noise is needed. In recent years, classification studies in areas such as music genre, speaker identification, and environmental sound have been actively conducted using machine learning [19–21]. In particular, the models and input features used in the field of environmental sound classification (ESC) can be used as a reference for this study. In the cases of EnvNet [22] and Sample-CNN [19], raw audio waveforms, which were not preprocessed, were used as inputs and classified with 1D convolution. There were many representations such as the mel-scaled spectrogram, mel-frequency cepstral coefficients, and log-power spectrograms. The convolutional neural network (CNN) proposed by Piczak [21], TFNet [23], and Multi-stream network [24] are models that use 2D CNNs for classification. Recently, despite the differences between image and audio features, a model to which the image domain CNN was applied demonstrated state-of-the-art performance [25, 26]. Palanisamy et al. applied the DenseNet, ResNet, and Inception models to the GTZAN, ESC50, and UrbanSound8K datasets, and DenseNet showed the best performance. Furthermore, it was found that when pretrained weights were used with ImageNet, the accuracy was 3%–20% higher than that of the model with randomly initialized weights [26]. Guzhov et al. proposed an ESResNet model based on the ResNet-50 architecture by adding a stack of attention blocks in parallel [25]. Research in ESC can be actively conducted because there are standard datasets, such as ESC50 [27] and UrbanSound8K [28], that enable a comparison between the performances of various models.

Inter-floor noise data are difficult to secure because of privacy issues. In addition, because the target is the sound transmitted to a lower level, the characteristics of the sound vary depending on room properties, such as the floor structure [29], shape of the room [30], and reverberation [31]. Moreover, when the noise level is not high, the signal-to-noise ratio may not be sufficient to produce reliable results. This study was conducted to confirm the possibility of an automatic classification system for inter-floor noise. An audio dataset obtained from recording in actual apartment houses in real living environments of the upper units was organized, and several CNN-based models were used for evaluation.

## Neighbor noise dataset

To establish a dataset for the classification of noise between floors, recording was performed for 24 h in three households that filed complaints owing to neighbor noise. The residents of the three households that were recorded and those of the upper floor households consented verbally to the use of the audio data for research purposes.

### Recording and annotation

**Fig 1:**
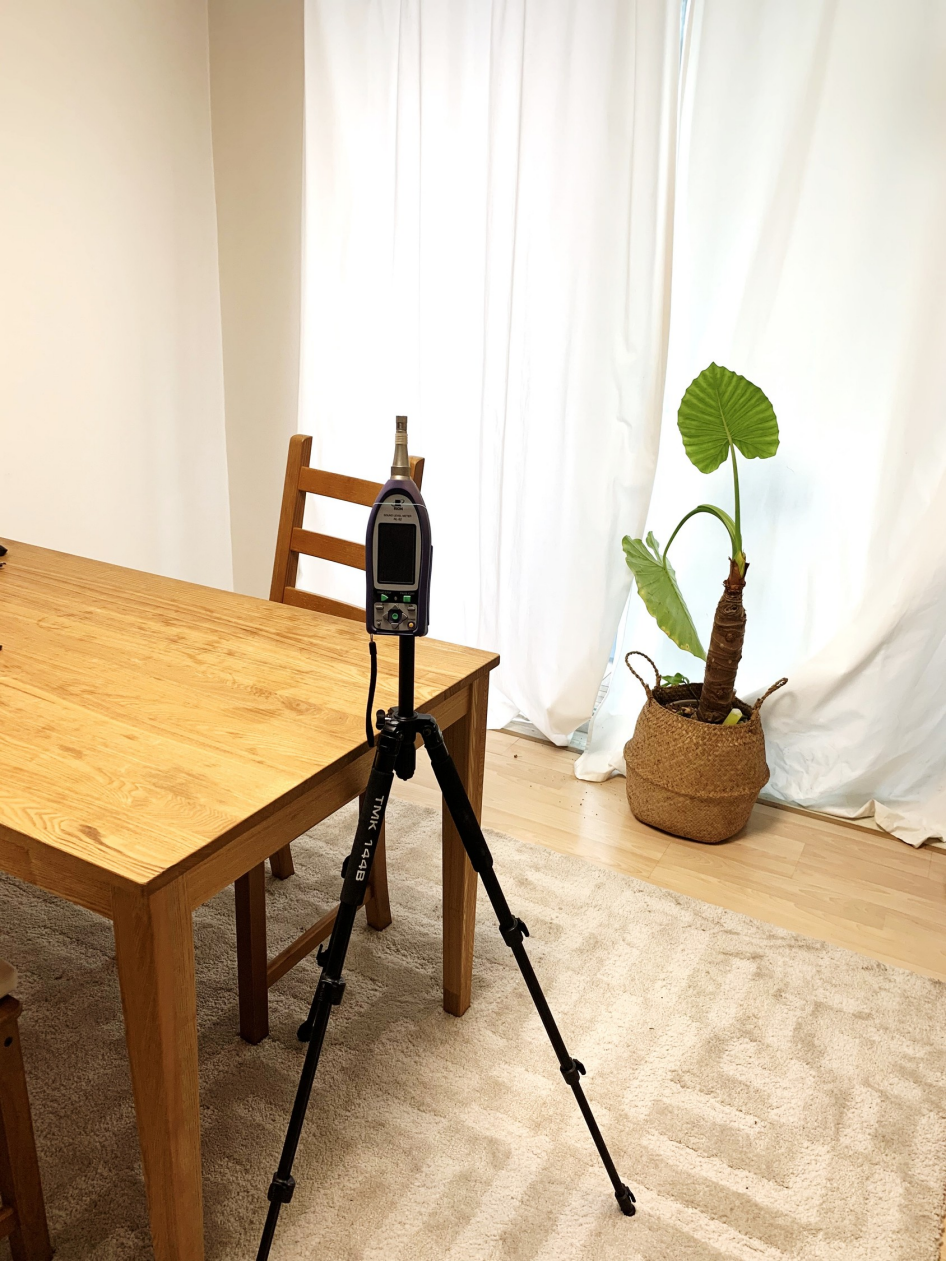
Inter-floor noise recording scene.

**Fig 2:**
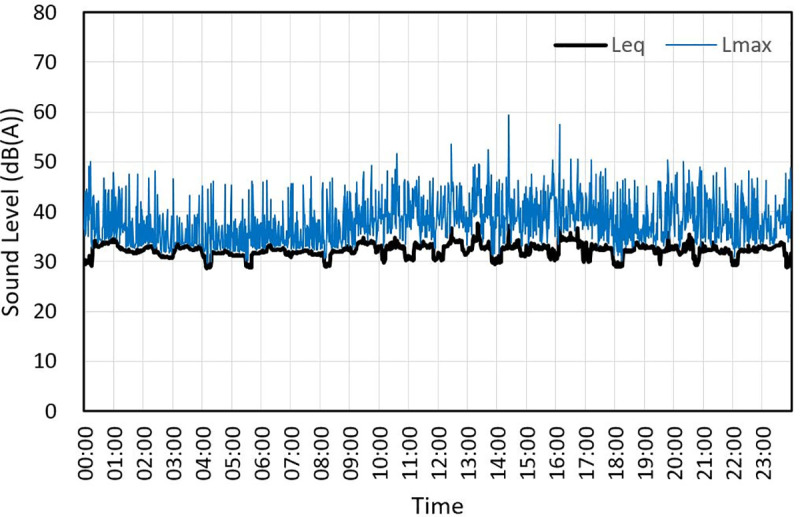
Sound level for 24 hours.

The recording was conducted with one microphone that was 1.5 m high in the center of the living room of the lower unit, as shown in Fig 1, and the residents in the upper unit lived as per their normal routine. To block external noise, windows of the lower unit were closed, and no one was present in these units. The recorded sound source for the section where equivalent noise level (L_eq_) exceeded 30 dB (A) during a 3-s section in the 24 h period of recorded data is shown in Fig 2. The annotators listened to the entire recording and indicated the source that generated the noise in the time section. While annotating the noise sources, the behavior of the upper unit was not monitored visually. This was because the sound may differ from that of ordinary days if the upper units’ residents’ behavior was filmed. The noise source determination was cross-checked with multiple annotators.

**Fig 3:**
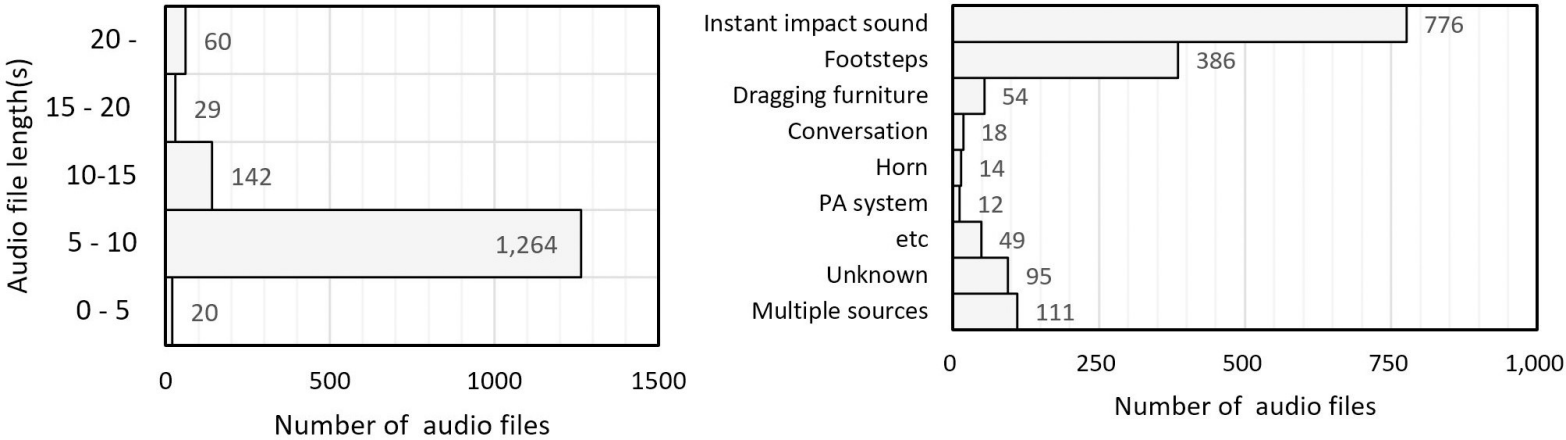
Recorded audio length and main sources.

The total number of sound sources recorded in three households for 24 h was 1,515. Fig 3 shows the length and main noise source of the recorded files, with most recordings being 5−10 s long. The number of noise sources is depicted in the following order: instant impact sound (dropping a heavy item), footsteps, dragging furniture, and conversation. A public announcement system (PA system) from the apartment management office and a car horn were also recorded, although these were not inter-floor noises. When the occurrence frequency was less than ten times, it was classified as “etc.”, and the sound of a cicada, dog barking, vacuum, and hammering were included. In some files with a longer recording time, noises from more than two noise sources were recorded (multiple sources); there were also sources that were difficult to distinguish (unknown).

### Neighbor noise dataset

**Fig 4:**
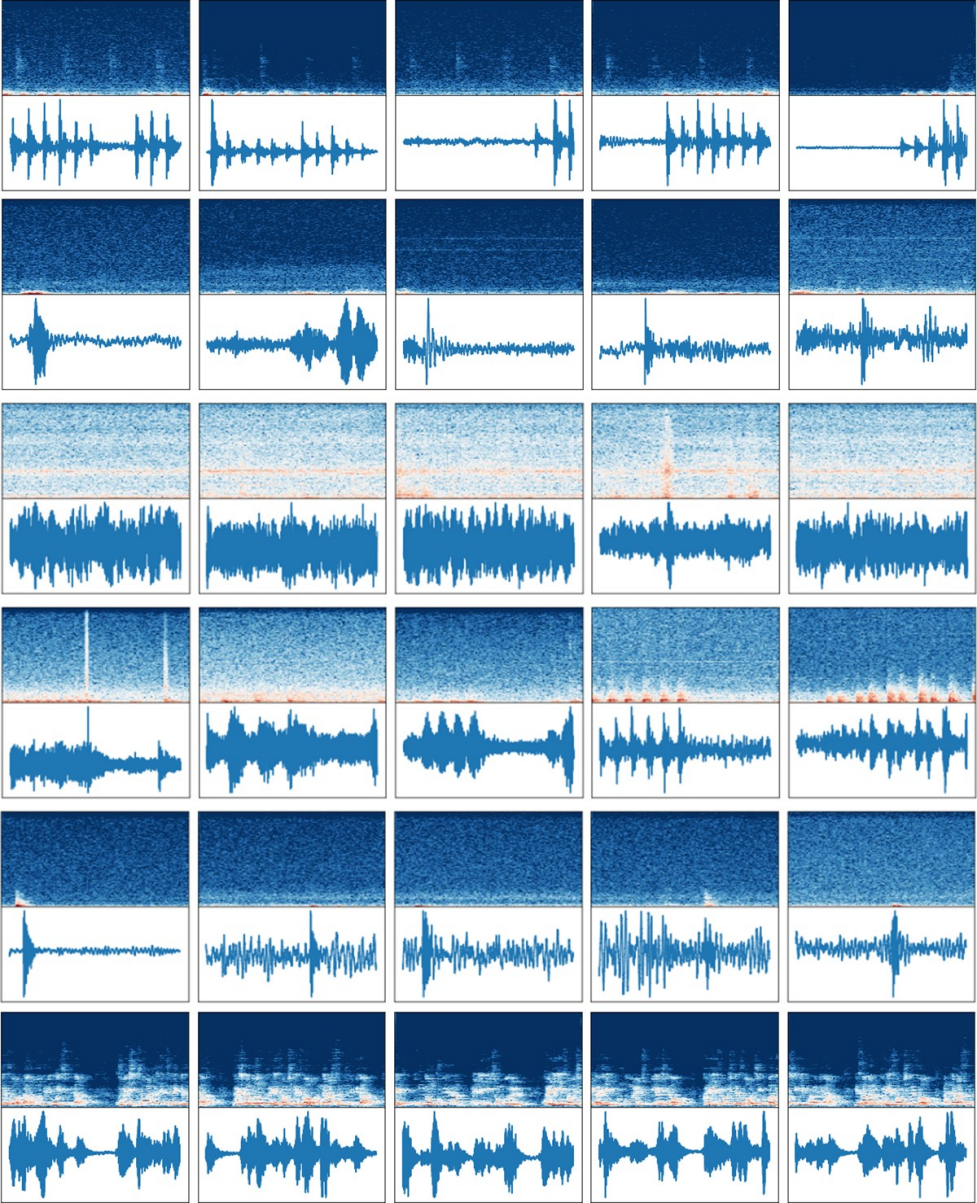
Waveforms and spectrograms. (a) Footsteps, (b) Dragging furniture, (c) Vacuum cleaner, (d) Hammering, (e) Instant impact and (f) PA system.

The instant impact, footsteps, dragging furniture, vacuum cleaner, hammering, and PA system were selected as the dataset classes considering the number of audio data, frequency of occurrence, and statistics of complaint [11], as shown in Table 1. In most files, there is no noise for 0-1s, and the time when the noise starts is approximately at 2−3 s. “Instant impact” and “Dragging furniture” as single noises were found to be mainly distributed between 2−5 s. Although the recording file was long for hammering, the noise occurred intermittently. The spectrograms and waveforms of the inter-floor noise dataset are shown in Fig 4.

**Table 1:** Inter-floor dataset configuration.

Category		Class	Description
**Indoor noise**	Noise from neighbors	Footsteps, Dragging furniture, Vacuum cleaner, Hammering, Instant impact	Inter-floor noise recorded in a lower unit 40 audio data per class
	Noise from own household	PA system	
**Exterior noise**	-	Helicopter, Chainsaw, Siren, Car horn, Engine, Train, Church bells, Airplane, Fireworks, Hand saw	“exterior/urban sound” category of ESC 50 audio dataset 40 audio data per class

In order to detect and classify the inter-floor noise in the recorded audio files, it is necessary to distinguish inter-floor noise from various other noises, such as household appliances noise and road traffic noises, which are also heard within a household. Therefore, this study intends to use a dataset that has been annotated in this study, combined with some parts of the ESC dataset. The ESC50 comprises five major categories: “animals,” “natural soundscape and water sounds,” “humans, non-speech sounds,” “interior/domestic sounds,” and “exterior/urban sounds.” Additionally, there are 10 classes with different sound sources for each category. Each class contains 40 audio clips that are 5 s long. Because many apartment buildings are exposed to traffic noises and construction site noises due to urban density [32], the audio dataset of the “exterior/urban sound” category was used. As shown in Table 1, this category consists of 10 classes of sound sources, such as helicopters, car horns, and sirens. The sample rate of the dataset was unified to 22,050 Hz, and the length was set to 4 s.

## Classification experiment

Models with high performance levels in image classification were used for the classification. The latest network engineering has improved its performance through the investigation of network elements such as depth, width, and cardinality [33]. Several models were designed to determine how to minimize the vanishing gradient problem, which occurs when the network depth is optimal. Skip connection was applied to ResNet [34], and a dense block was applied to DenseNet [35]. Inception [36] increases the width by applying several convolution filters. EfficientNet [37] is a recently proposed model. EfficientNet is a model that has improved its performance through compound scaling, which adjusts the width, scale, and image resolution. It has the highest accuracy for image classification tasks using ImageNet datasets and is an efficient model in terms of parameters and number of flops [37].

### Model setup

In this study, log mel spectrogram was used as a representation feature of audio. Based on Palanisamy et al. [26], the window size (25 ms, 50 ms, and 100 ms) and hop length (10 ms, 25 ms, and 50 ms) were calculated differently and entered into the three channels of the CNN model. To use the dataset efficiently, an augmentation of the training data was performed. Augmentation is a technique that can compensate for data scarcity and reduce overfitting. Augmentation was performed using methods such as time-stretching (controlling the speed of the audio without changing the pitch), pitch shifting (controlling the pitch without affecting duration), and background noise (mixed with white noise) [28], using Librosa library [38]. Furthermore, the weights pretrained with ImageNet were used as initial values. The learning rate was 1e-4 while the optimizer used was Adam with a batch size of 32 and NVIDIA Tesla P100 GPU accelerator. The number of epochs was 300. The performance was evaluated for accuracy, macro F1 score, macro precision, and macro recall through five cross-validations. Moreover, the computational complexity of each model was shown via the inference time. This performance was compared to the model showing state-of-the-art performance in audio classification.

## Experimental results

**Fig 5:**
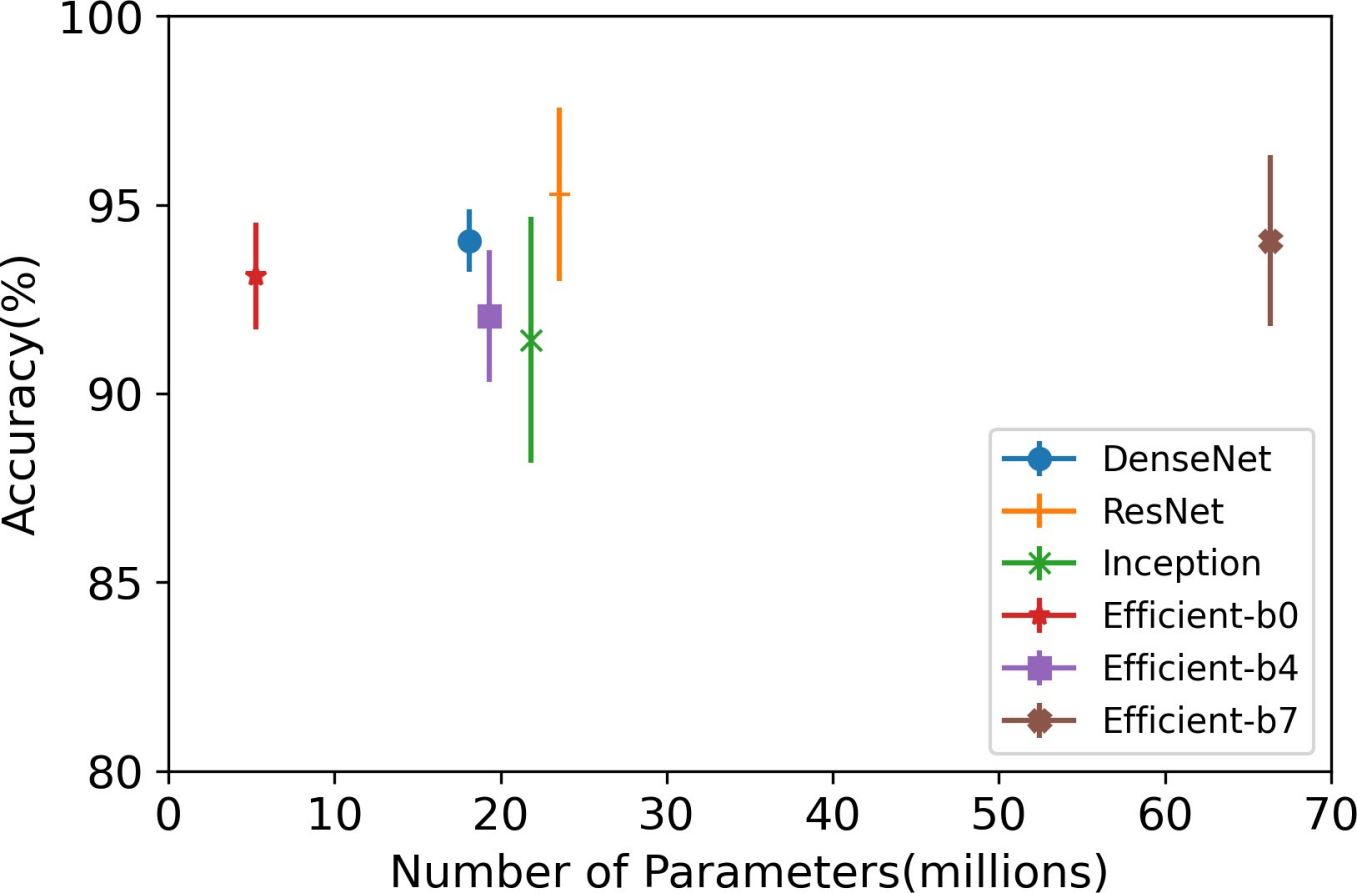
Model size and accuracy.

The dataset, including the inter-floor noise, was classified using CNN models, and the results are presented in Table 2. ResNet demonstrated the highest performance level for all metrics. In addition, ResNet showed the shortest inference time, despite having a larger network than the DenseNet and Inception models, as shown in Fig 5. After ResNet, DenseNet showed the next highest performance level. DenseNet showed the smallest performance deviation through a series of cross-validations. It means DenseNet is a more stable model with little difference in results regardless of the training data. Contrary to the expectations, the performance of EfficientNet was not superior to any other models. When EfficientNet classifies the ImageNet dataset, the accuracy also improves as the size of the model increases [37]. However, according to the results of the experiment in this study, EfficientNet-b0, which has the lowest number of model parameters, was found to have a higher performance than EfficientNet-b4 and b7.

**Table 2:** Classification results (5-fold cross validation).

Model	Performance metrics (Average±Standard deviation)				Inference time
	Accuracy	F1 Score	Precision	Recall	
**DenseNet**	94.05±0.83%	93.52±1.08%	94.45±0.83%	93.70±0.95%	30.3ms
**ResNet**	**95.27**±**2.30%**	**94.91**±**2.13%**	**95.51**±**2.03%**	**95.12**±**2.04%**	**6.2**ms
**Inception**	91.42±3.26%	90.84±3.06%	91.68±2.80%	91.21±2.88%	12.0ms
**E**fficientNet-B0	93.12±1.74%	92.63±1.56%	93.55±1.56%	92.81±1.65%	12.3ms
**E**fficientNet-B4	92.06±2.27%	91.43±2.28%	92.66±2.11%	91.73±1.99%	24.2ms
**E**fficientNet-B7	92.24±2.56%	91.87±1.96%	92.48±1.92%	92.00±2.03%	40.0ms

**Fig 6:**
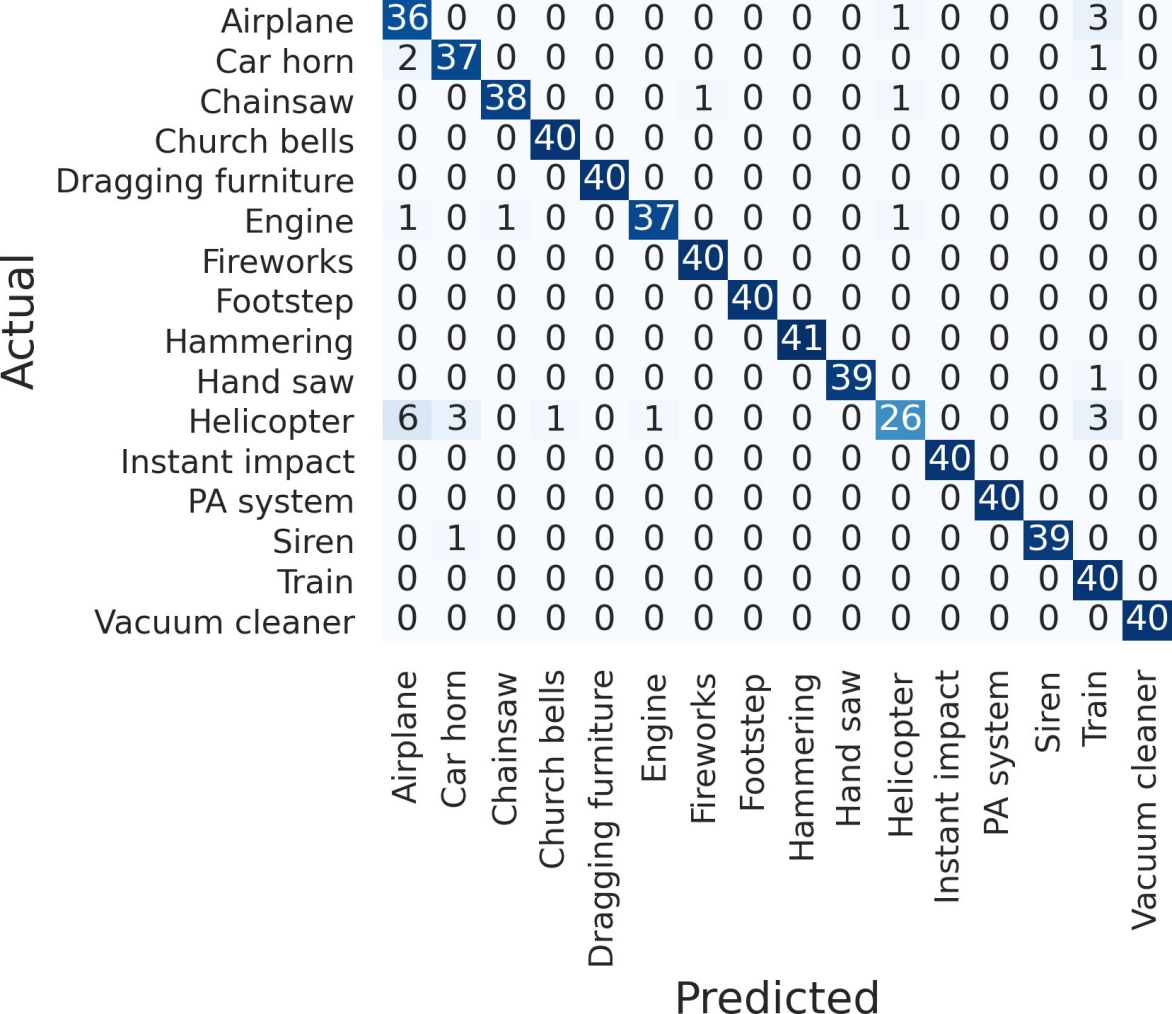
Confusion matrix of ResNet.

Fig 6 shows the confusion matrix of the ResNet classification. Compared to other sound sources among the datasets, “helicopter” was predicted as a different class; however, it did not affect the result of indoor noise classification. When classifying only inter-floor noise sources (i.e., dragging furniture, footsteps, hammer, instant impact, and vacuum cleaner), the accuracy of each class was 95%–100%.

The classification results of the study dataset and other audio datasets are presented in Table 3 [26]. In the case of GTZAN, ESC50, and Urban 8K, DenseNet exhibited a higher accuracy by 0.30–0.51% than ResNet. However, in the inter-floor dataset, it is necessary to examine why the performance of ResNet is higher. When the dataset was classified with pretrained weights using ImageNet, there was a 3%–20% performance improvement effect, as compared to when the initial weight value was random. Since this study also used the model of pretrained initial weights, it could attain an overall accuracy of 91.42–95.27%. In addition to ImageNet, when models trained with audio datasets such as GTZAN, ESC, and UrbanSound 8K were used, the initial weights were optimized for the audio domain.

**Table 3:** Comparison to other datasets.

Model	GTZAN		ESC50		UrbanSound 8K		Inter-floor noise
	Pretrained	Random	Pretrained	Random	Pretrained	Random	Pretrained
**DenseNet**	91.39%	88.50%	91.16%	72.50%	85.14%	76.32%	94.05%
**ResNet**	91.09%	87.90%	90.65%	67.40%	84.76%	73.26%	95.27%
**Inception**	90.00%	86.30%	87.34%	64.50%	84.37%	75.24%	91.42%
**EfficientNet**-b0	-	-	-	-	-	-	93.12%

For GTZAN, ESC50, Urban 8K classification results, refer to research by Palanisamy et al. [26].

Table 4 presents the ESResNet result [25], which is a state-of-the-art performance model. However, when compared with the ResNet, the accuracy was found to be 0.37% lower. Because the audio preprocessing methods of ResNet and ESResNet are different, they were needed to compare network performances with the same input features. To improve the performance of ResNet, Padam [39] proposed by Heo et al. was applied as an optimizer. When classifying music tagging, keyword spotting, and sound event tagging, Padam improved the accuracy by 0.18–0.80%, but with the dataset used in this experiment, the performance decreased by 1.29% when the optimizer was Padam. The inference time was reduced by 2.8 ms.

**Table 4:** Comparison to the state-of-the-art models.

Model	Performance metrics (Average±Standard deviation)				Inference time
	Accuracy	F1 Score	Precision	Recall	
**ResNet**	**95.27**±**2.30%**	**94.91**±**2.13%**	**95.51**±**2.03%**	**95.12**±**2.04%**	9.0 ms
**ESResNet** [25]	94.90±1.41%	94.57±1.44%	95.46±1.28%	94.67±1.38%	6.6 ms
**ResNet +Padam** [39]	93.98±2.06%	93.58±2.09%	94.53±1.58%	93.72±1.93%	**6.2 ms**

## Discussion

Although there is a demand for automation of detection and classification in inter-floor noise measurement services, there was no dataset available. In the case of urban noise, the performance of the developed model can be evaluated by using an annotated recording dataset, such as ESC-10, ESC-50, and UrbanSound8K. The classifier performance can be improved by comparing it with the existing models. To create such a dataset, one needs to record the actual noise, listen to the recorded data, and annotate the noise source, which is time consuming and costly. In this study, datasets including major neighbor noise sources, such as footsteps, dragging furniture, and hammering were organized. The inter-floor noise was classified using CNN models, including a state-of-the-art one. Among the models that were compared, ResNet showed the highest performance. The CNN-based model seems to be suitable for classifying 4s clips of a dataset. However, it is uncertain as to whether it is adequate for real-time monitoring, which requires the simultaneous processing of sequential data collected over a long time.

Although there are previous studies that classify the sources of inter-floor noise in datasets created through experiments [40], there is no study that detects footsteps based on actual inter-floor noise data. The automatic classification system can solve the problems of reliable replication of human noise source judgment and of privacy infringement. Currently, when one uses the noise measurement service, it costs approximately 600 USD. When the inter-floor classification is developed, a WAV file recorded by a user can be uploaded on the web or to the application, which enables one to use this service at 44 USD for a sound level meter [41]. Additionally, the system report can indicate whether the noise exceeded the legal limits, and the noise source, level, and generation time can be reported. Recently, IoT devices such as voice assistants are being actively used; these may serve as recording and noise monitoring tools.

In this study, 24 h of recorded data from three households were used. The recorded data included the inter-floor noise, external noise (road traffic noise and construction site noises), seasonal noise (cicadas and rain), and background noise (clock and ventilation system sound). To validate the reliability of the classifier, it must be tested in various acoustic scenes. In addition, a dataset was constructed for more diverse inter-noise sources, such as musical instruments, household appliances (TV, washing machine), conversation, and plumbing.

While constructing the inter-floor noise dataset, the actual behavior of a resident living in the upper unit could not be monitored. However, this is not considered to be a big issue because noise from the behavior of the upper unit resident, which is expected in the lower unit, has more effect on perception than the actual behavior. However, noise generated from the next unit or lower unit can also be transmitted [42]. Therefore, it is necessary to study the location of the noise generated in connection with the vibration sensor, to determine whether the sound detected by the inter-floor device has been generated by the behavior of the residents of the upper units.

The automatic classification model can also be used as a research method to study the effect on health, sleep, and soundscape of indoor space by monitoring the source, noise level, and generation time of inter-floor noise for prolonged periods. The demand for smart rooms is already increasing for security [43] and medical monitoring [44].

## Conclusions

In an early stage study of monitoring inter-floor noise in apartments, the present study organized a dataset with inter-floor noise recorded from three households under real living situations of upper units. The noise sources were classified using CNN models, and their accuracies were 91.43–95.27%. Among DenseNet, ResNet, Inception, and EfficientNet, the ResNet model showed the highest accuracy of 95.27±2.30% the highest performance in the metrics of F1 score, precision, and recall. Despite the large model network of ResNet, its inference time was the shortest. The accuracy of each class of inter-floor noise was found to be 95.0–100%. It is expected that this inter-floor noise detection and classification study will not only be applied to the inter-floor noise measurement service but also be used for research on soundscape recognition by identifying acoustic elements through long-term monitoring. In the future, the performance of the model should be verified according to various ranges of noise levels, signal-to-noise ratios, and background noises.
